# President’s Address at the Iowa and Nebraska Veterinary Medical Association1November 20, 1900.

**Published:** 1901-06

**Authors:** J. E. Brown

**Affiliations:** Oskaloosa, Iowa


### PRESIDENT'S ADDRESS AT THE IOWA AND NEBRASKA VETERINARY MEDICAL ASSOCIATION.[1]

By J. E. Brown, M.D.,
OSKALOOSA, IOWA.

Only a little over a year ago a number of Iowa and Nebraska veterinarians gathered here in this city to bear witness to and rejoice in the birth of a new veterinary medical society. This newcomer into the veterinary fraternity of the West was the outcome of a particular intimacy, friendship, and, finally, union in the interests of the veterinary profession of the veterinarians of these two States. Springing from such a parentage, we were naturally filled with enthusiasm and joyous anticipations concerning the future growth and development of the offspring. To-day we have reassembled to do her honor by the celebration of her first anniversary, and to plan for her future maturity and future usefulness. I am sorry that I am unable at the present time to refer by title to the object now claiming our attention, but unfortunately she has not yet been christened, the recommendation of a suitable name or title being one of the duties of our Committee on Organization not yet reported. There is apparently no limit to the growth and influence possible to this organization, nor to its value as a professional and social educator to the members of the Western veterinary profession. Here in the very centre of the great West —the greatest agricultural and stock-raising country that God ever permitted the sun to shine upon—it is certainly destined to become the garden-spot of the world for veterinary surgeons. Such a country will not only demand but will command the very best, the most learned and in every sense thoroughly competent and fully equipped veterinarians that the profession affords. The present representatives of the veterinary profession in the West are almost universally an energetic, hard-working lot of fellows, sufficiently ambitious to maintain their places in the front ranks, and to avail themselves of all the avenues leading to knowledge. It would be impossible for us to conceive of a more fertile source of practical knowledge than that which comes to us through a meeting of real, earnest, thoughtful, intelligent workers in veterinary science. Their consideration and discussions on the various subjects which come before them, the clinical demonstrations and individual intermingling one with another, are indeed ideal methods

[1] November 20, 1900.

for practical education. (Apparently some of our brethren in the rural districts have not yet learned this; but it is true, all the same.) Then, recognizing veterinary associations, through their meetings, as being the greatest propagators of real practical veterinary knowledge, we can only predict a wonderful growth for the one we are to-day to dedicate.

Our present organization is only a little nucleus around which will gather the wisdom and strength of the Western veterinary profession. Its influence shall manifest itself, and it shall be recognized throughout the length and breadth of the land on all great questions pertaining to veterinary and sanitary science.

While Iowa has not had nearly as many graduated veterinarians as many of the States further East, her State Veterinary Association has for many years enjoyed the reputation of being one of the best, both in point of attendance and in the character of its meetings.

Nebraska, with a far less number of veterinarians yet than Iowa can safely claim, I believe has a larger percentage of her veterinarians as members and supporters of her State Veterinary Association than any other State in the Union. It will now be seen that the veterinarians of these two States have assumed the responsibility of the support of three veterinary associations, and this in addition to the support they may and will give to the American Association.

Did I hear a voice dare to raise the question, "Will they support them?" Not much. Everybody knows too well the sort of stuff that these fellows are made of. They are not perfect; no, not that, but they do belong to that class that never say "die" when engaged in good work. We have our imperfections just like the balance of weak, struggling humanity. We have our shortcomings, which I believe, if we were duly reminded of from time to time, would work a wonderful improvement in our general condition. We do not see our own imperfections as others see them. Might I be pardoned, then, if I should call attention to some of them as they have appeared to me? One thing is the neglect of certain duties which we owe to the associations of which we are members. For many years, as many of you know, it has been my privilege to serve my own State association as secretary, and I speak with that knowledge that comes by the line of experience. As the time for our meeting approaches it becomes necessary to go among our members and solicit papers, reports of cases, etc. The excuses that have been offered in reply were simply astonishing. It was not that the members lacked interest exactly. They were interested in the success of the meetings, and so expressed themselves; but the illustration I wish to make is this: that the veterinarians, like the representatives of every other line of business here in the West, are so imbued with the spirit of hustle that it is only by an extreme effort that they can content themselves to quietly sit down and give their time and attention to such matters.

Now, gentlemen, let us not neglect such duties. Let each member bear in mind that he is jointly responsible for the success of the meetings. As we work along from time to time new ideas and new methods come to us, which when put into execution prove to be valuable aids in our work. Let me impress upon your minds the importance of making special notes of all such things for the express purpose of bringing them before just such meetings as this. Give the profession the benefit of your findings. Simple as some of these things may seem to you—too simple, you may think, to occupy the time of the meetings with—yet just remember that if the thought was new and good to you it will be new and good to someone else. I sometimes think that in these meetings such an effort is made to be what some might term scientific that many of the more practical and therefore helpful things are entirely overlooked. Do not be afraid to write papers or make up reports of certain interesting cases; either is always acceptable. Do not always wait to be asked to do these things. You can hardly imagine what a relief it is to have these come as volunteers. There are many more questions pertaining to association rights and privileges and duties that might advantageously be discussed. But I have in mind another subject which I consider of still greater importance to the future prosperity of the profession, so, after having started your minds along that channel of thought, it will be left for further consideration to your own good judgment. Too well do we all know how in the past every honest and conscientious effort that the veterinarians have made toward progression (I mean of a public nature) has been antagonized by that same old spirit, born of prejudice toward veterinarians, that has been so thoroughly grounded into the hearts of people by that uneducated, unintelligent, and usually morally degraded class of men who have been known in years gone by as "horse doctors." It has been found no easy task to overcome these prejudices, or to educate the public mind to distinguish the difference between the two classes. It was the lack of differentiation by the public that has been our constant chagrin and that has figured so conspicuously in the defeat of so many of the very laudable measures attempted by us which we well knew would mean a betterment in many of the conditions wherein the public would share the greatest benefits. It was the lack of respect, confidence, and influence in the profession by the public that has stood between us and our accomplishments of greatest public good. We become impatient and fret because we cannot eradicate these prejudices from the public mind more rapidly and command a greater influence, and I wonder if we ever stop to consider that one of the principal reasons why we cannot is the ever presence of "the wolf in disguise." They pose as qualified veterinarians and boast of diplomas as good as anybody's. We find them lounging about the streets, livery-barns, and race-tracks, and they manifest about the same degree of self-respect as do the men they find in such places and with whom they associate.

Gentlemen, do you realize that the one thing, greater than all others, that stands between us and that respect and influence which we would that we might command is that which savors of so much quackery and lack of self-respect—the careless habits and the unclean daily deportment to which so many veterinarians are slaves, yet so unfitted and unbecoming to professional men? Can we not, then, arouse within the hearts and minds of all our brethren a more determined effort toward the manifestation of the dignity that naturally should come with education and that always commands respect from all classes. I am not advocating absoluteness in this respect, except as it may apply to character and integrity, for there need never be any blight attached to that, for no one knows better than the speaker how difficult it is at all times and how impossible it is sometimes for a busy practitioner to maintain a presentable appearance; but, in the interests of the profession, I do earnestly plead for the observance of neatness and cleanliness in so far as it can be made compatible with our work. I am fully aware that of necessity with the dirty work we have to do our clothing cannot long be kept spotless—that is not expected—but there is no excuse for our habitually going about the streets, about our business, or appearing in public places in a careless, dirty garb and general slovenly make-up that we sometimes see. A respectable hat, a smooth-shaven face, a well-kept beard, a clean collar and tie, the actual mud and loose dirt brushed from the outer clothing, and a touch of polish on the shoes are features of everyday dress that go a long way toward giving one a respectable, gentlemanly appearance, and certainly none of these things is beyond the attainment of any practising veterinarian. It is true that very many of the people with whom we have to associate in business are not inspiring to us in this respect. We all know who they are. Many of them have come into the world blighted by birth ; they have no education, mentally or morally, that tends to inspire; hence no ambitions, and, therefore, with these there should be no comparison. I have not a word to utter against the honest laborers in the very lowest vocations of life; but certainly all veterinarians should aspire to rise above the style, the habits, and the associations of street loungers, the common hostlers, and the race-horse swipes. Yes, we must hold the confidence and respect even of these, yet in our work sometimes we would be in a measure handicapped; but gain and maintain that confidence and respect, not by constant companionship which breeds familiarity, but by kind words when thrown in contact, and ever gentlemanly conduct and always strict attention to our own business. It is one of the powers and privileges vested purely within ourselves that each may so deport himself that credit and honor will reflect not only upon himself, but also upon the profession which he represents, and, likewise, if he so deport himself as to reflect discredit and dishonor upon himself, the same will reflect discredit and dishonor upon the profession in the eyes of the people. Little do we realize sometimes the extent to which our moral and intellectual worth is estimated by those who see us, simply upon the evidence of our appearance and our general conduct.

Recapitulating these, if I am correct in the matter, the essential attributes to and on which the public will build confidence in our profession are, first, a proper education ; then self-respect, genteel habits and deportment, and an honest endeavor on the part of each of its representatives.

To obtain this of course no general law can be proclaimed, but let each of us adopt a moral standard such as we can conscientiously feel it should be, live up to and beyond it, and the examples we set will gradually but naturally be patterned after by those whom we have not yet been able to induce to come in and share in the good things of our meetings.

---

The next meeting of the Veterinary Medical Association of New Jersey will be held at Newark, July 11, 1901.

